# Reduced size at birth and persisting reductions in adiposity in recent, compared with earlier, cohorts of infants born to mothers with gestational diabetes mellitus

**DOI:** 10.1007/s00125-019-4970-6

**Published:** 2019-08-09

**Authors:** Philippa M. Prentice, Laurentya Olga, Clive J. Petry, David Simmons, Helen R. Murphy, Ieuan A. Hughes, Carlo L. Acerini, Ken K. Ong, David B. Dunger

**Affiliations:** 1 Department of Paediatrics, University of Cambridge, Box 116, Level 8, Cambridge Biomedical Campus, Cambridge, CB2 0QQ UK; 2 Present Address: Department of Paediatrics, North Middlesex University Hospital NHS Trust, London, UK; 3 Wolfson Diabetes and Endocrine Clinic, Cambridge University Hospitals NHS Foundation Trust, Cambridge, UK; 4 Present Address: Macarthur Clinical School, Western Sydney University, Sydney, NSW Australia; 5 Department of Diabetes and Endocrinology, Norfolk and Norwich University Hospital, Norwich, UK; 6 Women’s Health Academic Centre, Division of Women’s and Children’s Health, King’s College London, London, UK; 7 MRC Epidemiology Unit, Institute of Metabolic Science, Cambridge, UK; 8 Institute of Metabolic Science, University of Cambridge, Cambridge, UK

**Keywords:** Adiposity, Gestational diabetes mellitus, Growth, Infancy, Macrosomia, Maternal hyperglycaemia, Offspring, Skinfold thickness, Weight gain

## Abstract

**Aims/hypothesis:**

This study aimed to explore the infancy growth trajectories of ‘recent’ and ‘earlier’ offspring of mothers with gestational diabetes mellitus (OGDM), each compared with the same control infants, and investigate whether ‘recent’ OGDM still exhibit a classical phenotype, with macrosomia and increased adiposity.

**Methods:**

Within a prospective observational birth cohort, 98 ‘earlier’ OGDM born between 2001 and 2009 were identified using 75 g oral glucose tolerance testing at 28 weeks gestation, 122 recent OGDM born between 2011 and 2013 were recruited postnatally through antenatal diabetes clinics, and 876 normal birthweight infants of mothers with no history of diabetes were recruited across the full study period as the control group. All infants followed the same study protocol (measurements at birth, 3, 12 and 24 months, including weight, length and skinfold thickness indicating adiposity, and detailed demographic data). In all cases, GDM was defined using the International Association of Diabetes and Pregnancy Study Group criteria.

**Results:**

Earlier OGDM had higher birthweight SD scores (SDS) than control infants. Conversely, recent OGDM had similar birthweight- and length SDS to control infants (mean ± SD, 0.1 ± 1.0 and− 0.1 ± 0.9, respectively), but lower mean skinfold thickness SDS (−0.4 ± 0.6 vs 0.0 ± 0.9; *p* < 0.001). After birth, earlier OGDM showed reduced gains in weight and length between 3 and 12 months. In contrast, recent OGDM had increased weight and skinfold thickness gains until 3 months, followed by reduced gains in those variables from 3 to 12 months, compared with control infants. At 24 months, recent OGDM had lower adiposity than control infants (mean skinfold thickness SDS −0.3 ± 0.7 vs 0.0 ± 0.8; *p* < 0.001). At all time points recent OGDM had lower growth measurements than earlier OGDM.

**Conclusions/interpretation:**

Recent OGDM showed different growth trajectories to the earlier group, namely normalisation of birthweight and reduced adiposity at birth, followed by initial rapid weight gain but subsequent reduced adiposity postnatally. While avoidance of macrosomia at birth may be advantageous, the longer-term health implications of these changing growth trajectories are uncertain.

**Electronic supplementary material:**

The online version of this article (10.1007/s00125-019-4970-6) contains peer-reviewed but unedited supplementary material, which is available to authorised users.

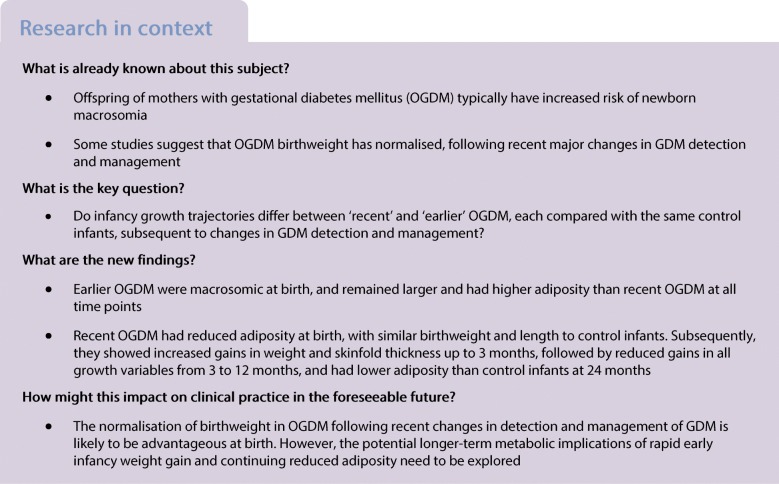



## Introduction

Offspring of gestational diabetic mothers (OGDM) are at increased risk of macrosomia [1] and higher newborn adiposity [2]. These physical traits are associated with obstetric and neonatal complications, including prematurity, shoulder dystocia, hypoglycaemia and jaundice [3]. Subsequently, OGDM demonstrate a relatively slow weight gain or ‘catch-down’ growth until 2 years of age [4, 5]. Weight gain is then accelerated after age 5 years [6] and linked to long-term increased metabolic risks, including obesity and type 2 diabetes [7–9]. However, in recent years, some studies suggest that birth size of OGDM may be normalising [10, 11].

This could be attributed to the changing diagnostic criteria for gestational diabetes mellitus (GDM) and subsequent more intensive management of gestational hyperglycaemia. In 2010, the International Association of Diabetes and Pregnancy Study Groups (IADPSG) suggested more stringent GDM diagnostic criteria than those used previously [12]. This followed results from the Hyperglycemia and Adverse Pregnancy Outcomes (HAPO) study, involving 23,000 non-GDM pregnant women across nine countries. The study found that maternal blood glucose was positively correlated with increased birthweight, cord blood C-peptide level, neonatal hypoglycaemia and Caesarean section delivery rates [13, 14]. Even before the implementation of these guidelines, several recent randomised controlled trials had shown decreased perinatal complications and more normal birthweights after intensive treatment of glucose intolerance in pregnancy (dietary advice, glucose monitoring, and insulin as needed), compared with routine pregnancy care [15–17]. The introduction of the IADPSG guidelines may lead to more mothers being treated and perhaps more intensively. The impact of these temporal changes in management on early infancy growth has not been documented. As well as large birthweight, low birthweight and rapid postnatal ‘catch-up’ growth may also have implications for future health [18].

We hypothesised that recent changes in the detection and management of GDM could have an impact on classical growth differences long-observed between OGDM and other infants. Within the context of an established prospective birth cohort, we retrospectively applied the 2010 IADPSG criteria to two groups of OGDM born in non-overlapping years and compared birth size and early infancy anthropometry in each group, against a control group unaffected by GDM.

## Methods

### Study population and design

The Cambridge Baby Growth Study (CBGS) has been recruiting newborns at the Rosie Maternity Hospital, Cambridge, UK for the study of pregnancy and postnatal determinants of early infancy growth and metabolism since 2001 [19]. Between 2001 and 2009 mothers were approached during pregnancy and all underwent a formal 75 g OGTT at 28 weeks gestation as part of a research protocol. The fasting and 2 h glucose results were fed back to guide clinical management.

Between 2011 and 2013, in addition to the recruitment of women with uncomplicated pregnancies, we specifically recruited mothers identified as having GDM from specialist antenatal clinics who had undergone a 50 g glucose challenge test followed by a formal OGTT.

For the purpose of the current analyses, the same IADPSG criteria were retrospectively applied both to those OGTT collected as part of research between 2001 and 2009 and to those carried out as part of the clinical diagnostic procedures between 2011 and 2013 in order to reduce any bias in severity of GDM resulting from changing diagnostic criteria over this period.

The retrospective application of the IADPSG criteria has implications for treatment as 19% of the ‘earlier’ OGDM group were not diagnosed and did not receive any treatment.

### Inclusion criteria

To select mothers with GDM for study of both cohorts (‘earlier’ and ‘recent’), IADPSG criteria [12] (at least one glucose concentration on a 75 g OGTT at around 28 weeks gestation: >5.1 mmol/l at 0 min, >10.0 mmol/l at 60 min, >8.5 mmol/l at 120 min) were applied retrospectively to the earlier cohort and prospectively in the recent OGDM group, allowing comparable earlier and recent OGDM subgroups. In all subgroups, the following additional criteria were met: singleton pregnancy; no significant maternal comorbidity (such as pre-eclampsia, hypertension, antiphospholipid syndrome, ankylosing spondylitis, lupus or ulcerative colitis); gestational age ≥36 weeks. Cases of maternal type 1 or type 2 diabetes and infants with a genetic or syndromal disease were excluded.

### GDM populations

The earlier OGDM (*N* = 98) were born between 2001 and 2009 and the recent OGDM (*N* = 122) were born between 2011 and 2013.

In the earlier OGDM population, a 75 g OGTT at 28 weeks gestation was performed as part of the research protocol and, for the purpose of the current study, the IADPSG criteria were applied retrospectively. In contrast, the clinical decision to treat these women in the earlier GDM cohort was broadly based on the WHO 1999 guideline [20], which considers fasting and 2 h glucose values (but not 1 h glucose). Based on available records and information from treating clinicians, GDM was mostly treated with diet and lifestyle modification, with or without insulin; metformin was not routinely used at that time.

In the recent OGDM population, women were recruited from the antenatal GDM clinic following routine practice (a 75 g OGTT in high-risk women and those identified through a universal 50 g glucose challenge at 24–26 weeks as a necessary prerequisite for the woman to undergo glucose tolerance testing). All women received standardised dietary and lifestyle advice, and were seen in clinic regularly (on average every 2 weeks). Additionally, metformin and/or insulin were prescribed as required, guided by regular fasting and postprandial glucose monitoring.

### Control population

A CBGS control population (*N* = 876) was comprised of mother–infant dyads with normal blood glucose levels on OGTT at 28 weeks using the IADPSG criteria between 2001 and 2009 (and those recruited later who had normal 50 g glucose challenge tests). They were studied using the identical postnatal research protocol.

Studies were approved by the Cambridge local research ethics committee, and all mothers gave informed written consent.

### Birth measurements and infancy anthropometry

Infants’ birthweights were obtained from hospital records. Newborn (within first 8 days) length and skinfold thickness, and subsequent measurements at 3, 12 and 24 months of age were performed by three trained paediatric research nurses, using identical protocols for all cohorts. Weight was measured to the nearest 1 g using a Seca 757 electronic baby scale (Seca, Birmingham, UK). Length was measured to the nearest 0.1 cm using an Infantometer (Seca 416). Skinfold thickness was measured in triplicate at four sites (triceps, subscapular, flank, quadriceps) on the left side of the body using a Holtain Tanner/Whitehouse Skinfold Caliper (Holtain, Crymych, UK).

### Statistical analyses

Infancy age- and sex-appropriate SD scores (SDS) were calculated for weight and length measurements (with adjustment for gestational age at birth and 3 months), by comparison with the UK 1990 growth reference using LMS growth software [21]. For each of the four skinfold thickness measurements an internal SDS was calculated, using residuals from a linear regression model, adjusting for infancy age, (gestational age at birth and 3 months) and sex. Mean skinfold thickness SDS was used in analyses. Maternal BMI was derived from self-reported pre-pregnancy weight divided by the square of measured height (kg/m^2^). Birth ponderal index was calculated by dividing the infant’s birthweight by its birth length cubed (kg/m^3^). Deprivation was assessed using an integrated index based on residential postcodes [22].

Maternal and birth characteristics were compared between groups using ANOVA with Bonferroni post hoc analysis for continuous variables, and χ^2^ tests for categorical outcomes. Unless otherwise stated, all data are presented as means ± SDs.

Multiple linear regression was used to investigate the effect of GDM on birth outcomes, allowing adjustment for potential confounders, including infant sex, postnatal age, gestational age, pre-pregnancy maternal BMI, maternal height, parity, breastfeeding history at 3 months, delivery method, maternal ethnicity, socioeconomic status reflected by Index of Multiple Deprivation (IMD) and pregnancy smoking history. All confounders were chosen a priori through the extensive work of CBGS and the Avon Longitudinal Study of Parents and Children (ALSPAC) [18].

Under the traditional listwise deletion method, only 68% of the control group and 64% of both recent and earlier OGDM had complete data on all covariates. Covariates with most missing values were maternal pre-pregnancy BMI for control infants and ‘earlier OGDM’, and smoking history for recent OGDM. Data were primarily missing due to incomplete perinatal questionnaire responses. Missing covariates including IMD (*n* = 3), parity (*n* = 4), maternal ethnicity (*n* = 8), smoking history during pregnancy (*n* = 39), maternal pre-pregnancy BMI (*n* = 185), maternal height (*n* = 148), delivery method (*n* = 27) and infant feeding history (*n* = 189) were imputed under the assumption that they are missing at random. The R package ‘Multiple Imputations via Chained Equations (MICE)’ was used to generate 20 imputed datasets, using normal linear regression for continuous variables and logistic linear regression for binary variables. Analyses run on each dataset were pooled according to Rubin’s rules [23]. Imputed values compared reasonably to observed values, and the results (i.e. linear regression model on birth data, Table 2) using listwise deletion were similar to imputed values, therefore imputed values were presented in the subsequent analyses.

In the visit measurements, missing data were commonly due to loss-to-follow-up or drop outs. In order to capitalise the longitudinal growth data with good handling of missing values, linear mixed-effects models were used to relate the continuous growth outcome variables (weight, height and skinfold thickness) to visit time point, cohort group, and their interaction with infant age, taking into account the same confounders as in the linear regression models for birth measurements. Due to non-linear relationships with age (indicated by significant estimates for age-squared), time was modelled using linear splines with knots at ages 3 and 12 months. Models were fitted to the data by restricted maximum likelihood (REML).

Statistical analyses were conducted using SPSS (IBM SPSS Statistics for Windows, version 25.0; IBM, Armonk, New York, USA) and R (version 3.3.2; R Foundation for Statistical Computing, Vienna, Austria). *p* < 0.05 was considered statistically significant.

## Results

### Women and pregnancy data

Table 1 shows the demographics of the women who participated in the study: 876 control, 98 earlier GDM and 122 recent GDM. Compared with the control group, women from the earlier GDM cohort had similar height, ethnicity and IMD levels. In contrast, women in the recent GDM cohort were shorter, more ethnically diverse, delivered at an earlier gestational age and were more deprived (with a higher IMD). Both GDM groups had higher BMI and increased smoking rate, compared with the control group.

**Table 1:** Maternal demographics, offspring birth characteristics and cross-sectional comparisons of infant growth variables

	Control (*N* = 876)	Recent OGDM (*N* = 122)	Earlier OGDM (*N* = 98)
Maternal demographics			
Age at birth (years)	33.4 ± 4.2	33.6 ± 5.1	33.4 ± 4.4
White	96%	76%*	98%
IMD	8.9 ± 3.3	11.3 ± 6.8**	9.1 ± 3.6
Primiparous pregnancy	48%	52%	37%*
Height (cm)	166.1 ± 7.2	162.7 ± 6.8**	165.8 ± 6.9
Pre-pregnancy BMI (kg/m^2^)	24.0 ± 4.4	27.0 ± 6.3**	26.6 ± 5.6**
OGTT gestational age (weeks)	28.5 ± 0.7	28.9 ± 5.6	28.5 ± 1.5
Fasting venous glucose (mmol/l)	4.2 ± 0.3	4.8 ± 0.8**	5.3 ± 1.1**
60 min venous glucose (mmol/l)	6.5 ± 1.4	10.6 ± 1.5**	9.2 ± 2.1**
120 min venous glucose (mmol/l)	6.0 ± 1.0	8.5 ± 1.6**	6.7 ± 1.9**
Smoking during pregnancy	3.8%	9.4%*	8.2%*
Offspring birth characteristics			
Gestational age (weeks)	40.0 ± 1.3	38.9 ± 0.9**	39.5 ± 1.4**
Caesarean delivery	28%	42%**	40%*
Male infant sex	52%	54%	53%
Weight (kg)	3.523 ± 0.481	3.303 ± 0.472**	3.632 ± 0.588
Weight SDS^a^	0.07 ± 0.93	0.10 ± 1.01	0.55 ± 1.13**
Length (cm)	51.5 ± 2.4	50.0 ± 2.0**	51.3 ± 2.7
Length SDS^a^	−0.05 ± 0.93	−0.07 ± 0.94	0.22 ± 0.97*
Ponderal index (kg/m^3^)	25.9 ± 3.2	26.3 ± 2.7	26.7 ± 3.2
Sum of skinfolds (mm)	24.6 ± 6.0	20.0 ± 3.6**	26.0 ± 6.3
Mean skinfold thickness SDS^a^	0.03 ± 0.86	−0.41 ± 0.61**	0.31 ± 0.85*
Macrosomia (birthweight >4.0 kg)	15%	7%*	27%**
SGA (birthweight <−1.5 SDS)	5%	4%	2%
Cross-sectional infant growth comparisons			
3 months	Total *n* = 710	Total *n*= 102	Total *n* = 91
Nutrition (exclusively breastfed)	45%	46%	38%
Weight SDS^a^	−0.05 ± 1.03	0.18 ± 1.04	0.29 ± 1.02
Length SDS^a^	0.14 ± 0.94	−0.03 ± 0.98	0.34 ± 0.96
Mean skinfold thickness SDS^a^	−0.01 ± 0.81	−0.07 ± 0.65	0.04 ± 0.78
12 months	Total *n* = 624	Total *n* = 86	Total *n* = 77
Weight SDS^a^	0.08 ± 1.07	−0.25 ± 1.26**	0.16 ± 1.00
Length SDS^a^	0.36 ± 1.09	−0.01 ± 1.07	0.31 ± 0.98
Mean skinfold thickness SDS^a^	0.02 ± 0.78	−0.36 ± 0.74**	0.21 ± 0.69
24 months	Total *n* = 611	Total *n* = 83	Total *n* = 76
Weight SDS^a^	0.19 ± 1.04	−0.03 ± 1.10	0.36 ± 0.96
Length SDS^a^	0.43 ± 1.05	0.34 ± 1.11	0.42 ± 1.11
Mean skinfold thickness SDS^a^	0.02 ± 0.83	−0.31 ± 0.65**	0.19 ± 0.64

Values are mean ± SD, or %

Weight-, length- and mean skinfold thickness SDS values are adjusted for gestational age, sex and postnatal age at measurement

Note: in control population, number of participants for length and skinfold thickness measurements at birth is 573

Cross-sectional infant growth comparisons are adjusted for sex, postnatal age at measurement, pre-pregnancy maternal BMI, maternal height (for length only), parity and 0–3 months feeding history. Comparisons at age 3 months are additionally adjusted for gestational age at birth

^a^SDS for weight and length are calculated using the UK 1990 reference, for skinfold thickness using internal references

*p < 0.05 vs control group

**p < 0.005 vs control group

Recent GDM women were also significantly different from earlier GDM women: they were shorter, delivered at an earlier gestational age, were more likely to have primiparous pregnancies, and had lower fasting but higher OGTT 60 min venous glucose concentrations.

### Infants’ anthropometric data

Earlier OGDM were significantly heavier at birth (Table 1), as expected, even after further adjustment for all covariates (Table 2, *p* = 0.01). Fully adjusted birthweight and adiposity of earlier OGDM were significantly greater than those of recent OGDM (Table 2).

**Table 2:** Linear regression comparison of infant growth variables at birth between groups

Outcomes		Recent OGDM vs controls (controls as reference)		Earlier OGDM vs controls (controls as reference)		Recent vs earlier OGDM
		β ± SE	*p*	β ± SE	*p*	*p*
Weight SDS	Model 1	−0.11 ± 0.09	0.24	0.21 ± 0.05	<0.0001	0.001
	Model 2	−0.05 ± 0.1	0.629	0.16 ± 0.05	0.002	0.01
Length SDS	Model 1	−0.01 ± 0.11	0.925	0.08 ± 0.06	0.192	0.157
	Model 2	−0.11 ± 0.11	0.306	0.05 ± 0.06	0.347	0.567
Skinfold SDS	Model 1	−0.51 ± 0.09	<0.0001	0.07 ± 0.05	0.167	<0.0001
	Model 2	−0.53 ± 0.09	<0.0001	0.05 ± 0.05	0.385	<0.0001

**β** (regression coefficients) ± SE are displayed

Model 1: adjusted for gestational age (birth and 3 months growth outcomes only), sex and age at measurement

Model 2: Model 1 + adjusted for pre-pregnancy maternal BMI, maternal height (for height gain only), parity (primiparous, yes/no), feeding history (exclusively breastfed at 3 months, yes/no; except for birth anthropometry), maternal ethnicity (white, yes/no), IMD, delivery method (Caesarean delivery, yes/no), maternal smoking history during pregnancy (yes/no)

Skinfold, skinfold thickness

**Fig. 1:**
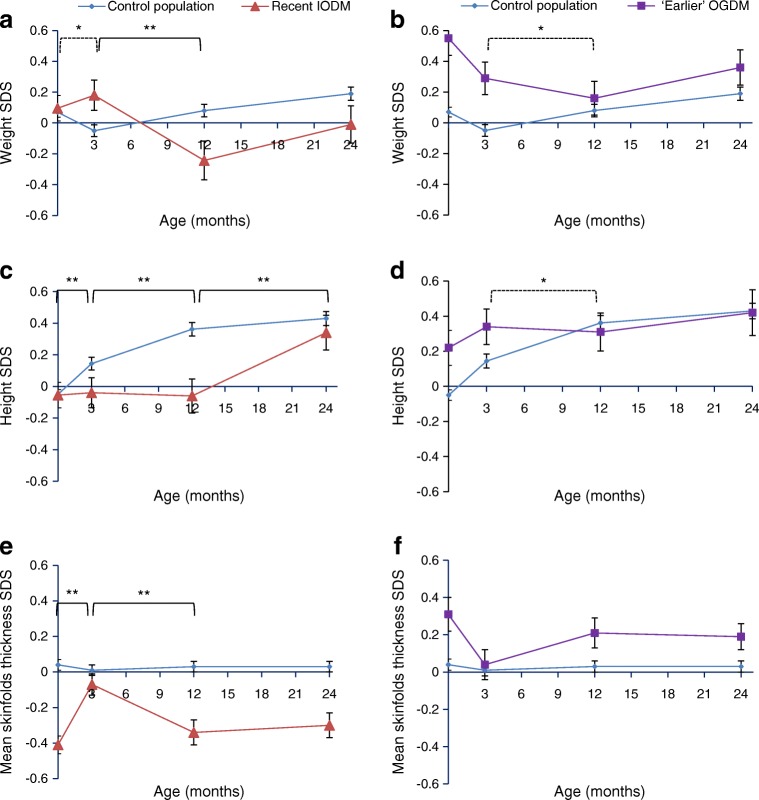
(**a**–**f**) Weight, length/height and skinfold thickness growth trajectories of recent or earlier OGDM compared with control infants from birth to 2 years. Plotted values are mean ± SEM, adjusted for sex, gestational age (birth and 3 months only), and age at measurement. Comparisons are adjusted for sex, postnatal age at measurement, pre-pregnancy maternal BMI, maternal height (for length/height only), parity, feeding history at 0–3 months, delivery method, maternal ethnicity, socioeconomic status reflected by IMD and pregnancy smoking history. Comparisons between 0 months and 3 months are additionally adjusted for gestational age at birth. Horizontal bars indicate statistically significant differences between OGDM and control groups for the displayed growth periods (* and dashed bar: *p* < 0.05; ** and solid bar: *p* < 0.001). Significance is based on linear mixed-effect models of infant growth variables between groups, with time modelled using linear splines (Table 3)

After birth, earlier OGDM showed significant downwards growth trajectories in weight and length between 3 and 12 months compared with control infants (Fig. 1, Table 3). Between 12 and 24 months, weight, height and skinfold thickness of earlier OGDM followed a comparable growth trajectory to control infants. However, at all time points, earlier OGDM maintained slightly higher mean skinfold thickness compared with control infants.

**Table 3:** Linear mixed-effect models of infant growth variables between groups

Outcomes		Recent OGDM vs controls (controls as reference)		Earlier OGDM vs controls (controls as reference)		Recent vs earlier OGDM
		Estimate±SE	*p*	Estimate±SE	*p*	*p*
Change 0–3 months						
Weight SDS	Model 1	0.07 ± 0.03	0.03	−0.05 ± 0.04	0.18	0.0096
	Model 2	0.07 ± 0.03	0.039	−0.05 ± 0.04	0.172	0.01
Length SDS	Model 1	−0.06 ± 0.03	0.04	−0.03 ± 0.03	0.372	0.477
	Model 2	−0.06 ± 0.03	0.035	−0.03 ± 0.04	0.36	0.467
Skinfold SDS	Model 1	0.14 ± 0.03	1.37 × 10^−5^	−0.04 ± 0.04	0.277	8.43 × 10^−5^
	Model 2	0.14 ± 0.03	1.54 × 10^−5^	−0.05 ± 0.04	0.235	6.45 × 10^−5^
Change 3–12 months						
Weight SDS	Model 1	−0.06 ± 0.01	1.39 × 10^−7^	−0.03 ± 0.01	0.01	0.06
	Model 2	−0.06 ± 0.01	1.42 × 10^−7^	−0.03 ± 0.01	0.01	0.057
Length SDS	Model 1	−0.03 ± 0.01	0.004	−0.03 ± 0.01	0.009	0.943
	Model 2	−0.03 ± 0.01	0.005	−0.03 ± 0.01	0.01	0.969
Skinfold SDS	Model 1	−0.03 ± 0.01	0.009	0.02 ± 0.01	0.15	0.0025
	Model 2	−0.03 ± 0.02	0.008	0.02 ± 0.01	0.146	0.002
Change 12–24 months						
Weight SDS	Model 1	0.015 ± 0.01	0.123	0.003 ± 0.01	0.768	0.368
	Model 2	0.01 ± 0.01	0.127	0.003 ± 0.01	0.79	0.361
Length SDS	Model 1	0.029 ± 0.005	2.78 × 10^−5^	0.004 ± 0.01	0.638	0.007
	Model 2	0.035 ± 0.008	2.95 × 10^−5^	0.004 ± 0.009	0.688	0.006
Skinfold SDS	Model 1	0.004 ± 0.01	0.638	−0.005 ± 0.01	0.602	0.455
	Model 2	0.004 ± 0.01	0.649	−0.006 ± 0.01	0.578	0.446

Fixed effect estimates (visit period and group interaction) ± SE are displayed

Smoothing splines were added to the models with knots at 3 and 12 months

Model 1: adjusted for gestational age (birth and 3 months growth outcomes only), sex and age at measurement

Model 2: Model 1 + adjusted for pre-pregnancy maternal BMI, maternal height (for height gain only), parity (primiparous, yes/no), feeding history (exclusively breastfed at 3 months, yes/no; except for birth anthropometry), maternal ethnicity (white, yes/no), IMD, delivery method (Caesarean delivery, yes/no), maternal smoking history during pregnancy (yes/no)

Skinfold, skinfold thickness

In contrast, recent OGDM had similar birthweight and length to control infants, but lower adiposity (skinfold thickness SDS **β** ± SE: −0.53 ± 0.09, fully adjusted model, *p* < 0.0001) (Table 2). All individual skinfolds were significantly lower but triceps, flank and quadriceps skinfold thickness were particularly reduced (electronic supplementary material [ESM] Table 1).

After birth, recent OGDM showed different growth trajectories to the earlier OGDM and the control group. From birth to 3 months, weight and skinfold thickness gains were significantly increased compared with those of control infants and earlier OGDM, but with reduced length gain compared with the control group (Fig. 1, Table 3). This was followed by reduced gains in weight, length and adiposity between 3 and 12 months, compared with control infants (Fig. 1, Table 3). Weight and length SDS for recent OGDM became comparable with that of control infants only at 24 months, reflecting increased growth between 12 and 24 months. However, their skinfold thickness remained lower than that of control infants even at 24 months (Fig. 1).

## Discussion

This observational study demonstrates significant differences in both birth size and subsequent infancy growth trajectories between recent and earlier OGDM compared with control infants. Recent OGDM had comparable birthweight and length SDS, but unexpectedly reduced skinfold thickness, indicating lower adiposity, compared with control infants. Conversely, earlier OGDM were heavier at birth, consistent with the traditional description of OGDM [1, 2].

Some recent studies concur with our findings. An Australian study (2013) [10] found no significant difference between recent OGDM and control infants’ birthweights; and a UK study (2016) [11] reported lower weights and lengths for OGDM vs control infants at 2 weeks of life. Recent GDM trials (no treatment vs lifestyle advice +/− insulin) also suggest a shift towards the normal population distribution of birthweights in OGDM [16, 17].

Reduced subcutaneous fat at birth in our recent OGDM may be a novel finding. Au et al [10] demonstrated lower body fat percentage in OGDM at birth, (7.9 ± 4.5% vs 9.5 ± 4.3% in the control group), but this was not statistically significant in their relatively small study (*N* = 67) [24]. Conversely, a recent systematic review and meta-analysis published in 2017 concluded that newborn adiposity is still increased in OGDM [25]. However, although the overall numbers in that analysis were large, individual studies were small, and many combined type 1 diabetes, type 2 diabetes and GDM. Two of the most recent studies showed no body fat percentage difference between OGDM and control infants [26, 27]. Logan et al [11] found no difference in total adipose tissue mass on MRI in OGDM compared with control infants at 11 days of age. Therefore, our study and recent literature suggest that GDM diagnosed and treated over the last decade may result in offspring birthweight and length comparable with the general population, and even reduced adiposity.

Our recent OGDM cohort showed significantly increased weight and skinfold thickness gains compared with the control group from birth to 3 months, despite similar breastfeeding rates. A comparable UK cohort (2011–2014) also found greater weight and adiposity gains from birth to 2.5 months in OGDM [11]. However, in contrast with our findings, their OGDM cohort still had greater total adipose tissue at 2.5 months, adjusting for sex and maternal pre-pregnancy BMI [11]. Our recent OGDM then showed reduced gains in weight and skinfold thickness from 3 to 12 months, resulting in significantly reduced weight and adiposity at 12 months compared with control infants. Subcutaneous adiposity in this group remained lower than in the control group until 24 months of age. In contrast, earlier macrosomic OGDM showed expected catch-down growth, with slightly reduced weight and skinfold thickness gains from birth to 3 months, and significantly decreased gains in weight and length between 3 and 12 months, compared with control infants. However, they still had higher adiposity than control infants at all time points.

The smaller birth size seen in recent OGDM was evident despite higher maternal BMI and higher OGTT 60 min glucose concentrations compared with earlier OGDM. Both groups were retrospectively defined using the IADPSG criteria. Therefore the normalisation of birth anthropometry seen in recent OGDM is probably due to intensification of GDM monitoring and treatment, rather than inclusion of individuals with ‘milder’ GDM. We hypothesise that this could result from tighter glycaemic control per se, direct effects of medication transported across the placenta, or interactions between these environmental factors, genetic predisposition and epigenetic modulation. Conversely, earlier OGDM showed predicted increased birthweight, due presumably to greater nutrient supply in pregnancy and fetal hyperinsulinism. A weakness of our study is that we do not have uniform data on glucose variability, maternal treatments and HbA_1c_ to further inform this debate.

Amelioration of the classic macrosomic phenotype is likely to be associated with fewer adverse outcomes; however, the long-term effect of early reduced infancy weight and subcutaneous fat could be associated with risk itself, particularly if leading to catch-up growth, which has been associated with risk for childhood obesity and adult type 2 diabetes [18, 28]. The finding that tight glucose control can not only normalise birthweight but also be associated with reduced body size has been previously reported. Langer et al investigated three GDM groups and showed that the group with lowest maternal glucose values had a higher proportion of small for gestational age (SGA) infants [29]. The timing of treatment could also play a role as a recent study reported that early GDM treatment was associated with a higher rate of SGA-related neonatal intensive care unit admissions, whereas later treatment resulted in more large for gestational age infants [30]. The recent HAPO data relating to the follow-up of infants born to mothers with a wide range of glucose values at 28 weeks gestation confirm a positive relationship between those levels and adiposity at 10–14 years (skinfold thickness and air displacement plethysmography) [31]. As well as linking high antenatal glucose exposures to childhood overweight/obesity, we could also infer from these data that lower glucose exposures might result in persisting reduced adiposity. Therefore, while there are clear advantages of intensive multidisciplinary GDM management, there may also be negative implications for some OGDM.

In addition to more extensive diet and lifestyle advice, medical treatment of GDM has changed significantly over recent years [11]. Metformin is now commonly used worldwide, often as first-line medication, and crosses the placenta in significant amounts. While we were unable to include medication use in our analyses, 20% of women were treated with metformin (+/− insulin) during recent GDM recruitment, compared with near zero for the earlier GDM (clinic data). We therefore postulate that metformin itself may at least partly explain the differences seen between recent and earlier OGDM anthropometry and growth trajectories. The Metformin in Gestational Diabetes (MiG) trial, randomising women with GDM to metformin (+/− insulin if needed) or insulin, suggested that metformin might affect infancy fat deposition patterns. There were no differences at birth [32]. However, at 2 years of age, children from the metformin group had increased subscapular and biceps skinfold thickness, despite no difference in overall fat, suggesting a more favourable fat distribution [32]. Our recent OGDM cohort with greater metformin exposure, compared with the earlier group, shows preferentially increased gains in 3 month subscapular skinfold thickness and then reduced gains until 2 years of age (ESM Table 1).

At 7–9 years, OGDM randomised to metformin in the MiG trial had similar total body fat and metabolic measures, although the 9 year olds were larger [33]. A study in polycystic ovary syndrome (PCOS) suggested a growth restriction effect of metformin in infants of normal-weight mothers [34]. It is therefore hard to interpret whether metformin may confer a beneficial fat distribution or an increased long-term risk of obesity. Metformin effects may also differ depending on maternal weight gain, glycaemic control and other environmental factors. Further studies are needed to elucidate the effect of metformin itself, effects on maternal energy intake and weight gain, and interactions with other environmental factors, on adiposity distribution.

To our knowledge, no previous study has investigated growth trajectories of recent OGDM, in comparison with a control group, up to 24 months of age. Strengths of this study include measures of length and skinfold thickness adiposity, in addition to weight, in a large cohort. Collection of detailed maternal and demographic data allowed adjustment for potential confounding factors. Use of the IADPSG diagnostic criteria [12] means that our results are relevant to populations worldwide, where GDM is now diagnosed using lower thresholds and is more aggressively treated. The ‘recent’ OGDM cohort showed a slightly increased prevalence of ethnic minority groups. However, ethnicity was not a significant covariate in our growth models. Local hospital demographic data (not shown) for all GDM mother–infant dyads born at the same time as the recent group were similar to the study population, and therefore anthropometric findings of recent OGDM at birth were unlikely to result from study participation bias. Furthermore, the anthropometric measures for more recent control participants recruited in Cambridge have not shown any evidence of a secular trend in infancy growth (data not shown).

Limitations of the study include that the two OGDM groups are not fully comparable, and no details are available of glycaemic control after GDM diagnosis, although it is likely that more intensive treatment of recent GDM women led to tighter glycaemic control. Since data regarding weight gain during pregnancy were incomplete, it cannot be confirmed that recent GDM women had adequate pregnancy weight gain. The study population was large compared with most previous studies; however, there were insufficient numbers to investigate the individual effects of metformin and insulin on anthropometric outcomes. A further limitation is that 19% of women in the earlier cohort were identified retrospectively and did not receive GDM treatment. However, excluding these women would still give similar outcomes in the regression models (ESM Table 2). Studies are needed to further understand the mechanisms responsible for anthropometric outcomes in OGDM, and ideal GDM treatment going forward. We believe that the trend has been towards stricter application of ‘targets’ blood testing and the greater use of metformin. A weakness of our study is that we do not have accurate details of glucose concentrations, HbA_1c_ or metformin use in these populations. Going forward, continuous glucose monitoring data defining individual glucose exposures may clarify these factors. Our work and that of others suggests that it will be informative to further study adiposity distribution in OGDM, including subcutaneous and visceral fat deposits, to investigate beneficial vs undesirable adiposity gains.

It is debatable whether reduced early anthropometric measures in OGDM will have positive or negative implications, particularly for longer-term health. In the neonatal period it may be advantageous, allowing normal birthweights and reduced pregnancy complications. However, this could result in increased numbers of SGA infants, and associated comorbidities. Reduced size at birth, leading to subsequent early catch-up growth, may also lead to later increased metabolic disease risk [35].

## Conclusion

We hypothesise that recent changes in the detection and intensive multidisciplinary management of GDM, with dietary and lifestyle modifications, and medication where needed, has improved glycaemic control resulting in a birth size similar to control infants. The normalisation of birthweight, compared with earlier OGDM, may be associated with improved later-life metabolic health [36]. However, our findings and those of others show subsequent increased anthropometric gains, akin to postnatal catch-up growth in SGA babies, which may convey its own metabolic risks [28]. It may be that there will be a trade-off between tighter glycaemic control, direct effects of medication, birth size and infancy growth trajectories. This cohort continues to be followed up and later childhood data will be of interest, as well as replication in other populations.

## Electronic supplementary material

ESM 1 (PDF 136 kb)

## Data Availability

Data are available on request from the authors.

## Abbreviations

CBGS: Cambridge Baby Growth Study
GDM: Gestational diabetes mellitus
HAPO: Hyperglycemia and Adverse Pregnancy Outcomes
IADPSG: International Association of Diabetes and Pregnancy Study Groups
IMD: Index of Multiple Deprivation
MiG trial: Metformin in Gestational Diabetes trial
OGDM: Offspring of gestational diabetic mother
SDS: SD score
SGA: Small for gestational age
