# Supplementary material for: Reduced size at birth and persisting reductions in adiposity in recent, compared with earlier, cohorts of infants born to mothers with gestational diabetes mellitus
Source: Diabetologia. 2019 Aug 9;62(11):1977–87. doi: 10.1007/s00125-019-4970-6 (PMC6805804; doi:10.1007/s00125-019-4970-6)
Supplement: Supplementary file 1 — (PDF 136 kb) [file 125_2019_4970_MOESM1_ESM.pdf]

ESM Table 1. Comparison of individual skinfold thickness SDS (internally derived) between ‘recent’ or ‘earlier’ OGDM versus controls

	Control	‘Recent’ OGDM	‘Earlier’ OGDM
**Birth**	**Total n=780**	**Total n=122**	**Total n=91**
Triceps	0.04 ± 1.03	-0.42 ± 0.65**	0.30 ± 0.97*
Subscapular	0.02 ± 1.02	-0.22 ± 0.87*	0.17 ± 1.01
Flank	0.05 ± 0.97	-0.59 ± 0.64**	0.45 ± 1.26**
Quadriceps	0.04 ± 1.03	-0.40 ± 0.66**	0.32 ± 1.01*
**3 months**	**Total n=710**	**Total n=102**	**Total n=91**
Triceps	0.02 ± 1.04	-0.27 ± 0.75*	0.18 ± 0.92
Subscapular	-0.01 ± 1.00	0.14 ± 0.98	-0.09 ± 1.01
Flank	0.00 ± 1.03	-0.03 ± 0.83	0.01 ± 0.92
Quadriceps	0.01 ± 1.03	-0.13 ± 0.77	0.09 ± 0.97
**12 months**	**Total n=621**	**Total n=86**	**Total n=77**
Triceps	0.03 ± 1.02	-0.47 ± 0.85**	0.27 ± 0.88
Subscapular	0.01 ± 1.02	-0.09 ± 0.97	0.05 ± 0.90
Flank	0.03 ± 1.01	-0.38 ± 0.80**	0.24 ± 1.04
Quadriceps	0.04 ± 1.00	-0.50 ± 0.90**	0.29 ± 0.98
**24 months**	**Total n=710**	**Total n=102**	**Total n=91**
Triceps	0.02 ± 1.03	-0.28 ± 0.87*	0.16 ± 0.88
Subscapular	0.01 ± 1.04	-0.13 ± 0.82	0.07 ± 0.81
Flank	0.03 ± 1.04	-0.43 ± 0.68**	0.26 ± 0.79
Quadriceps	0.03 ± 1.02	-0.44 ± 0.78**	0.25 ± 0.92

Values are mean ± SD, or %
SDS, standard deviation score (internally derived)
*p<0.05 vs. control group
**p<0.005 vs. control group
Comparisons are adjusted for sex and postnatal age at measurement. Comparisons at birth and age 3 months are additionally adjusted for gestational age.

ESM Table 2. Linear regression comparison of infant growth parameters between 'Earlier' OGDM and controls, with/without untreated subjects.

Model 1: adjusted for gestational age (birth and 3 months growth outcomes only), sex, and age at measurement
Model 2: Model 1 + adjusted for pre-pregnancy maternal BMI, maternal height (for length and height gain only), parity (primiparous, yes/no), feeding history (exclusively breastfed at 3 months, yes/no; except for birth anthropometry), maternal ethnicity (Caucasian descent, yes/no), index of multiple deprivation, delivery method (Caesarean delivery, yes/no), maternal smoking history during pregnancy (yes/no).

β (regression coefficients) ±SE are displayed

**Outcomes**			**'Earlier' OGDM vs. Controls**	
			**β±SE**	***p***
At birth				
Weight SDS	Model 1	Include all subjects	**0.27±0.06**	***<0.0001***
		Without 19% untreated subjects	**0.26±0.06**	***<0.0001***
	Model 2	Include all subjects	**0.15±0.06**	***0.01***
		Without 19% untreated subjects	0.12±0.07	*0.064*
Length SDS	Model 1	Include all subjects	**0.14±0.06**	***0.015***
		Without 19% untreated subjects	**0.13±0.06**	***0.048***
	Model 2	Include all subjects	0.08±0.06	*0.201*
		Without 19% untreated subjects	0.05±0.07	*0.462*
Skinfolds SDS	Model 1	Include all subjects	**0.13±0.05**	***0.015***
		Without 19% untreated subjects	**0.16±0.06**	***0.005***
	Model 2	Include all subjects	0.02±0.06	*0.699*
		Without 19% untreated subjects	0.05±0.06	*0.382*
Change 0-3 months				
Weight SDS	Model 1	Include all subjects	-0.10±0.06	*0.085*
		Without 19% untreated subjects	-0.12±0.07	*0.079*
	Model 2	Include all subjects	-0.06±0.07	*0.338*
		Without 19% untreated subjects	-0.05±0.07	*0.529*
Length SDS	Model 1	Include all subjects	-0.06±0.05	*0.2*
		Without 19% untreated subjects	-0.085±0.05	*0.117*
	Model 2	Include all subjects	-0.07±0.05	*0.205*
		Without 19% untreated subjects	-0.097±0.06	*0.107*
Skinfolds SDS	Model 1	Include all subjects	-0.12±0.07	*0.073*
		Without 19% untreated subjects	**-0.16±0.07**	***0.024***
	Model 2	Include all subjects	0.003±0.07	*0.967*
		Without 19% untreated subjects	-0.03±0.08	*0.671*
Change 3-12 months				
Weight SDS	Model 1	Include all subjects	-0.1±0.06	*0.07*
		Without 19% untreated subjects	-0.11±0.06	*0.079*

	Model 2	Include all subjects	-0.07±0.06	*0.234*
		Without 19% untreated subjects	-0.08±0.07	*0.251*
Length SDS	Model 1	Include all subjects	**-0.14±0.06**	***0.015***
		Without 19% untreated subjects	**-0.15±0.06**	***0.017***
	Model 2	Include all subjects	-0.09±0.06	*0.13*
		Without 19% untreated subjects	-0.07±0.07	*0.31*
Skinfolds SDS	Model 1	Include all subjects	**0.13±0.06**	***0.023***
		Without 19% untreated subjects	**0.14±0.06**	***0.034***
	Model 2	Include all subjects	0.1±0.07	*0.123*
		Without 19% untreated subjects	0.11±0.07	*0.147*
Change 12-24 months				
Weight SDS	Model 1	Include all subjects	-0.01±0.04	*0.8*
		Without 19% untreated subjects	-0.01±0.04	*0.803*
	Model 2	Include all subjects	-0.02±0.04	*0.702*
		Without 19% untreated subjects	-0.01±0.04	*0.758*
Length SDS	Model 1	Include all subjects	0.01±0.04	*0.9*
		Without 19% untreated subjects	0.01±0.04	*0.779*
	Model 2	Include all subjects	0.03±0.04	*0.541*
		Without 19% untreated subjects	0.03±0.05	*0.524*
Skinfolds SDS	Model 1	Include all subjects	-0.03±0.05	*0.5*
		Without 19% untreated subjects	-0.04±0.06	*0.486*
	Model 2	Include all subjects	-0.05±0.06	*0.379*
		Without 19% untreated subjects	-0.05±0.06	*0.453*
